# Use of Hangeul Twitter to Track and Predict Human Influenza Infection

**DOI:** 10.1371/journal.pone.0069305

**Published:** 2013-07-24

**Authors:** Eui-Ki Kim, Jong Hyeon Seok, Jang Seok Oh, Hyong Woo Lee, Kyung Hyun Kim

**Affiliations:** 1 Department of Biotechnology and Bioinformatics, College of Science and Technology, Korea University, Sejong, Korea; 2 Department of Electronics and Information Engineering, College of Science and Technology, Korea University, Sejong, Korea; National University of Singapore, Singapore

## Abstract

Influenza epidemics arise through the accumulation of viral genetic changes. The emergence of new virus strains coincides with a higher level of influenza-like illness (ILI), which is seen as a peak of a normal season. Monitoring the spread of an epidemic influenza in populations is a difficult and important task. Twitter is a free social networking service whose messages can improve the accuracy of forecasting models by providing early warnings of influenza outbreaks. In this study, we have examined the use of information embedded in the Hangeul Twitter stream to detect rapidly evolving public awareness or concern with respect to influenza transmission and developed regression models that can track levels of actual disease activity and predict influenza epidemics in the real world. Our prediction model using a delay mode provides not only a real-time assessment of the current influenza epidemic activity but also a significant improvement in prediction performance at the initial phase of ILI peak when prediction is of most importance.

## Introduction

Influenza is an important respiratory infectious disease causing seasonal epidemics or occasional pandemics across the world with considerable morbidity and mortality. Much of the observed wintertime increase of mortality in temperate regions is attributed to seasonality of influenza which is easily spread by airborne droplets made when an infected person coughs, sneezes or talks. Surveillance has become important to detect clusters of influenza cases and to focus public health resources on mitigating the spread and impact of the outbreaks. However, tracking the spread of an epidemic influenza in populations is a difficult task. Information on surveillance systems used to routinely monitor influenza activity such as influenza-like illness (ILI) has been collected to estimate the relative severity of influenza seasons [1]. Although ILI surveillance provides a valuable picture of influenza activity, ILI reports come directly from doctors and other health service professionals by 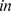 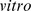 identification of influenza viruses, typically with a delay of up to one to two weeks. Since Google search engine query data were detected to be closely associated with seasonal influenza activity [2], there has been growing interest in monitoring influenza outbreaks using other digital media [3], [4].

Twitter is a social networking service that enables its users to exchange text-based messages of up to 140 characters known as tweets, including Hangeul (the Korean language). Useful information for tracking or even forecasting behavior when extracted in an appropriate manner lies embedded in the Twitter stream. Twitter has been used for a variety of purposes in many fields of human activity. It was shown that monitoring the contents of the Twitter messages can improve the accuracy of detecting models by providing early warnings of influenza outbreaks [5]. While Google tracking was found highly correlated with ILI statistics over a long time period [2], Twitter messages can provide more descriptive information than search engine query data, and estimates of ILI derived from the media can accurately track influenza activity [3], [4]. Twitter has over 500 million active users as of 2012, generating over 340 million tweets daily and handling over 1.6 billion search queries per day [6]. Although Twitter appears to be targeted to a young generation, it has attracted a diverse set of users in terms of age. The majority of Twitter’s nearly 10 million visitors in February 2009 were 35 years or older, and a nearly equal percentage of users were between ages 55 and 64 as were between 18 and 24 [7]. In August 2012, the demographic breakdown on the social network still reveals that most users in Twitter and Facebook are 35 or older, and the average Twitter and Facebook users are 37.3 and 40.5 years old, respectively [8].

Hangeul as one of the most perfect phonetic system devised [9] is the native alphabet of the Korean language, which consists of 24 consonant and vowel letters. Unlike the letters of the Latin alphabet, Hangeul letters which are grouped into blocks are shaped similar to the features of the sounds they represent. We have collected over 287 million Korean tweet messages for a 51 week period from October 2011 to September 2012. In this study, we have examined the use of information embedded in the Hangeul Twitter stream to detect rapidly-evolving public awareness or concern with respect to influenza transmission, and developed regression models that tracked levels of actual disease activity and can predict the ILI activity level in a population using a delay mode.

## Methods

### Twitter Stream Data Collection

In order to analyse public concerns regarding influenza activity, Hangeul tweets containing influenza-related words were collected from Twitter GardenPipe stream [10] beginning April 2011 via Query class in twitter4j that was an unofficial Java library for the Twitter application programmers interface (API) from TWITTER4J.org [11]. Collection of tweets were expanded via FilterQuery beginning October 2011. We developed JAVA-based Twitter timeline collector and the filtered Twitter stream constituted a subset of the entire stream [3]. To preserve the integrity of the collected tweets, we included tweets containing Hangeul characters only. Due to the limit of word counts in FilterQuery, data collection process was mainly based on a set of 190 frequently used and pre-filtered single characters and 10 infectious disease names in Hangeul.

We excluded tweets of less than five characters and re-tweets which contained less than two characters before first “RT @” or those starting with “RT @”. Advertisement sentences that contained “[“and”]” marks, hyper-linked sentences that contained “http://” string, sentences from robotic Twitter users, blank-ignored tweets, and spam tweets that cause too much messaging from one source were removed. Valuable re-tweets and tweets, if they were considered to be redundant of Tweet ID, were also removed. Tweets were then stored in Oracle 10g database, after removing approximately 20% of the collected tweets. Since the search on the Oracle database became significantly slow as the amount of data increased, the texts with search strings were stored in the Oracle database.

### Generation of Marker Frequency Matrix

Influenza is still often confused with the common cold but influenza symptoms usually are more severe than the typical sneezing and stuffiness by a cold. For this reason, keywords which have obvious connection to influenza or common cold were used to extract all the tweet messages containing them. The extracted messages were segmented into individual words divided by blanks, which were ranked as the most frequently used words: e.g. a total of 500 words were considered as initial marker corpus (Table S1). As the words were analysed for selecting markers, they were found to contain phonological and morphological features including homonyms to the term of influenza, honorifics that could be confused with influenza, and words with the same stem. As an example of phonological and honorific features in 500 most common words, [c^h^u.u.ni.ka], [c^h^u.un], [c^h^u.w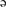], [c^h^u.wi], [c^h^up.go], [c^h^up.ne], [c^h^up.da], [c^h^up.sp.ni.da], [c^h^up. jo], and [c^h^up. i] came from [c^h^up.da] or [c^h^u.wi] which means the word “COLD”. The words including these phonological features and misspells were eliminated and those with the same stem were assigned to the same word. From this set of influenza related markers, we generated a daily marker frequency matrix from the Twitter corpus of a day.

Let the set of tweets on the 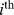 day be 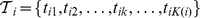 where 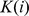 is the total number of tweets collected on the day. The frequency of the 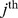 marker on the 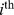 day, 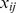, is defined as
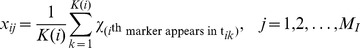
(1)where 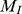 is the number of markers and 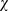 is an indicator function



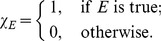



### Processing of ILI Data and Marker Selection by LASSO

We used ILI daily reports from Korea Centers for Disease Control and Prevention (KCDC) as a disease spreading reference. KCDC also provided regional statistics for influenza, which included the number of ILI patients among 1,000 visits reported by the Korea Influenza Surveillance Scheme [12]. The ILI activity was calculated by the number of ILI over the total number of inpatients (per thousand). For use of stationary estimation, the unreported ILI activities were filled by linear interpolation of the previous and next ILI activities to better express a weekly tendency in the ILI data, since KCDC’s daily ILI report is not available on Sundays or holidays. Baseline level of ILI activities in daily surveillance influenza outbreak was found to exhibit a threshold of 0.5 persons out of 100. In addition, the missing data in marker frequency matrix were also estimated by the same procedure, in order to remove the effect of spurious noise and periodic components, probably introduced by the weekly work pattern. The resulting marker frequency is further processed by applying a 7-point moving average on each column of the frequency matrix 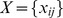. For the remainder of the paper, whenever we refer to ILI data and marker frequency they are interpolated and smoothed data.

After interpolation and smoothing, we selected a subset of markers for daily estimation by using the LASSO (Least absolute shrinkage and selection operator) algorithm. LASSO has an effect of automatically performing marker selection by using a single tuning parameter to control both the marker selection and the shrinkage component of the fitting procedure [13].

### Linear Regression

Daily influenza spreading score was estimated with both ILI and selected marker frequency matrix by using linear least squares regression algorithm, where the model coefficients were chosen to minimize the error metric or residual sum of squares [14]. Data set was created using Microsoft Excel format and numerical methods were applied as follows. Let.


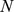
: number of days for which KCDC’s ILI data and Twitter data are available


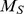
: number of selected markers


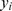
: KCDC’s ILI data for the 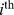 days, *i = 1, 2,…, N* (smoothed using 7-point moving average)


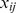
: frequency of marker 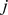 on the 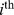 day (smoothed using 7-point moving average)

The linear estimator of 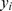, 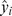, is



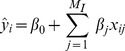
(2)where 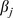
’s are the coefficients of regression. The residual, the error of the linear estimator, is



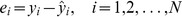
(3)The residual sum of squares (RSS) which is a function of vector of regression coefficients 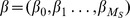 is



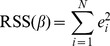
(4)We find the optimal coefficient vector 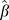 by minimising RSS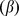. That is,
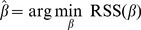
(5)


The optimal coefficient vector 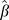 is obtained by solving a system of (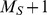) linear equations
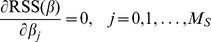
(6)


### ILI Prediction Algorithm

**Figure 1:**
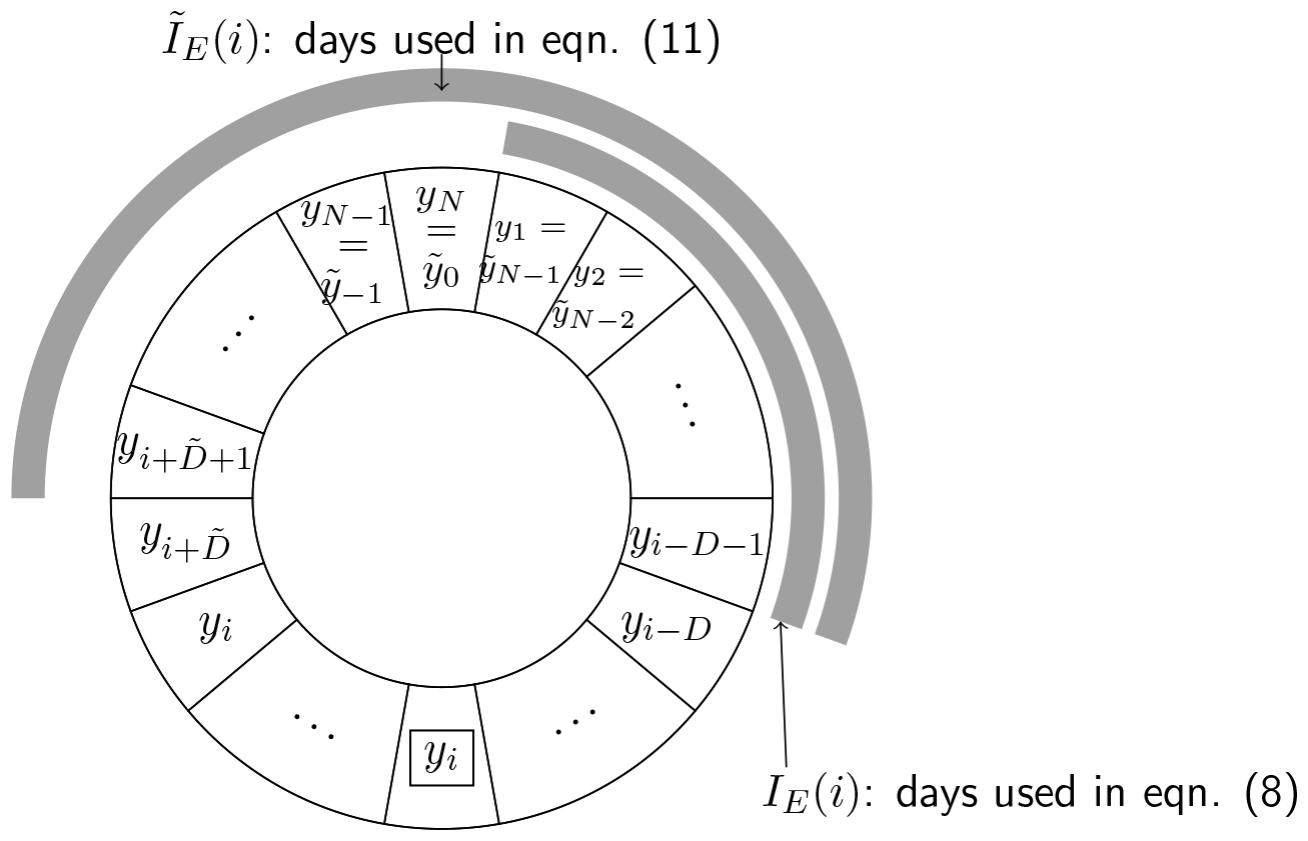
Subset of days used for regression coefficients for 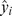. 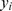
 is only available on the 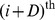
day.

We assumed that KCDC’s ILI data for the 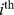 day is reported after 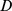 days of delay (Figure 1). That is, 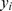 is only available on the 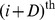 day. We attempted to determine the coefficients of the linear regression model based on the past data available for predicting ILI of the most recent days. In this context, when we computed 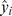 using equation (2), we needed to compute the regression coefficients using 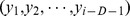 and 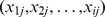 for 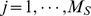.

The following algorithm, called Prediction Algorithm 1 (PA1), was a natural modification of the linear estimation described by equations (2)–(6).

**PA1**

For 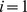 to 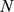, we performed (a)-(c).

(a) Let the subset of days 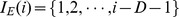 and
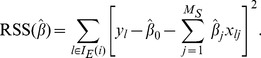
(7)where, 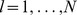. Here 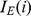 is the days when KCDC’s ILI is available for the computation of 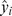 (Figure 1).

(b) Obtain 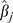 for 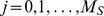 by solving a set of 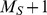 equations
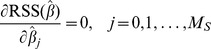
(8)


(c) Compute 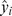 by
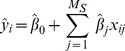
(9)


The limitation of PA1 is that during the initial period of 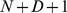 days of an influenza season, we do not have enough data to compute the coefficients of the linear regression. Even if there are enough data for the computation of the regression coefficients, the model obtained using small amount of available data poorly predict 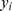
’s especially when there is a significant time variation of influenza activity. We believe that this problem can be partially resolved if the data for the previous influenza season were used for the computation of the regression coefficients. However, the present data we have is limited to only one influenza season. We, therefore, duplicated the avaiable data and used it as if it were the data of the previous influenza season. In computing the regression coefficients, we excluded the data corresponding to not only 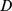 days of reporting delay but also 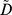 days after the post black-out period as shown in Figure 1. This is reasonable under the assumption that the Twitter users’ behaviour does not vary significantly from season to season.

The modified prediction algorithm, called Prediction Algorithm 2 (PA2), is given as follows. Here, we use tilde to denote the extension of the ILI and marker frequency matrix into the past (Figure 1).

**PA2**

For 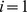 to 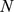, we performed (a)–(c).

(a) Let the subset of days 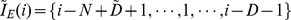 and
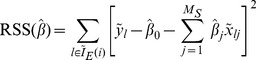
(10)


where



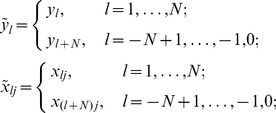



and 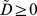 is the length of blackout period for estimating 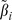
’s.

(b) Obtain 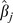 for 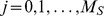 by solving a set of 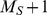 equations
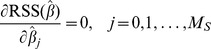
(11)


(c) Compute 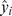 by
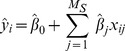
(12)


## Results

### Markers and their Correlations

**Figure 2:**
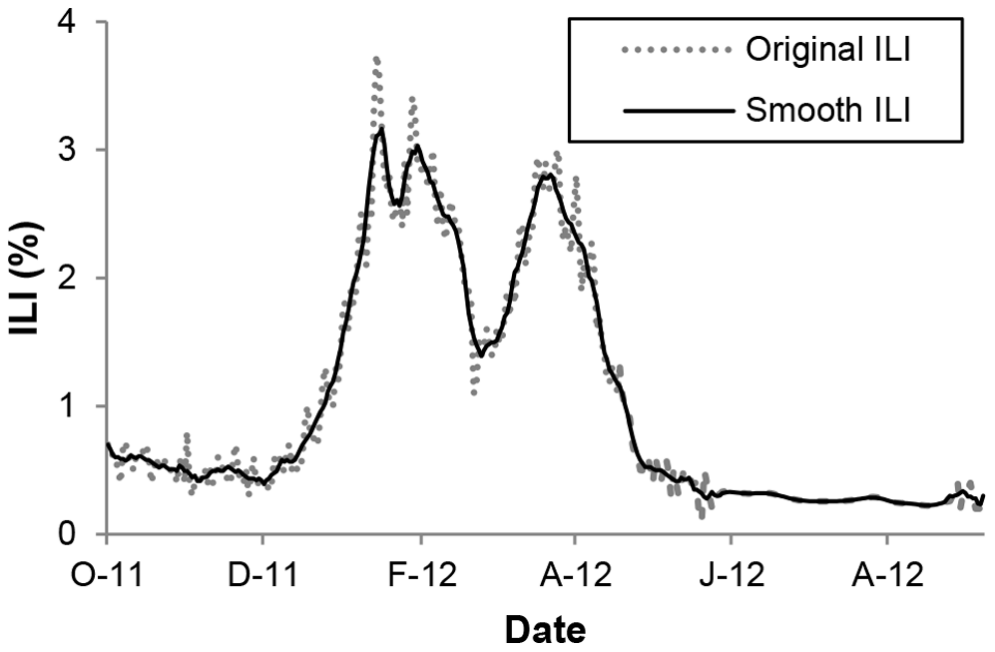
Comparison of original and smoothed ILI data by KCDC. The data were observed between October 2011 and September 2012.

The data set in our database consists of 881 thousand tweets containing influenza-related keywords, influenza and common cold, which were selected from 287 million Hangeul tweet timelines observed between October 2011 and September 2012. The size of the data set represented over 0.3% of the entire tweet volume. Interpolation and smoothing of ILI and marker frequencies seem to give a reasonable compromise between time resolution and rejection of high frequency noise as shown in Figure 2. The original and smoothed ILI activities by KCDC suggest that the smoothed ILI captures the important peaks of the ILI while removing unwanted noise. Interestingly, the frequencies of many of the markers were found to be not only weakly correlated with KCDC’s ILI data but also highly correlated with those of at least one of the other markers.

We initially made use of subjective markers derived from keywords which have direct connection to influenza. A pool of selected markers were then extracted from the 500 most common words which were found to form a very good description of the topic as well as many irrelevant ones. In order to rank weights for the selected markers, their regression coefficients were calculated. Inspecting the selected markers of the model revealed large positive coefficients assigned to markers like ‘novel flu’ (0.853), ‘flu’ (0.843), ‘severe’ (0.673), and ‘influenza’ (0.638). However, the algorithm also selected the terms ‘furthermore’ and ‘lightly’, which do not have any obvious connection to influenza. The term ‘lightly’ occurred frequently in phrases like ‘Since I dressed lightly, I caught the flu’ which was a common expression for catching a flu. The selected markers included not only illness symptoms but also irrelevant terminologies such as ‘likely’, ‘concert’, ‘due to’, and ‘for mercy’s sake’.

In order to examine the nature of data, the correlation of the selected markers with the ILI data was first examined. Let ILI data from KCDC be represented by a vector 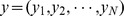 and the frequency of the 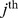 marker be by a vector 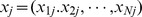, and define.


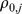
 = correlation between ILI data 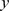 and the frequency of the 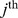 marker 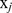.


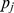
 = 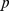-value of the 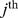 marker.


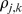
 = correlation between the 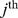 marker frequency 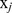 and the 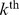 marker frequency 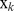.

Here, 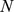 is the number of days when KCCD’s ILI data is available.

**Figure 3:**
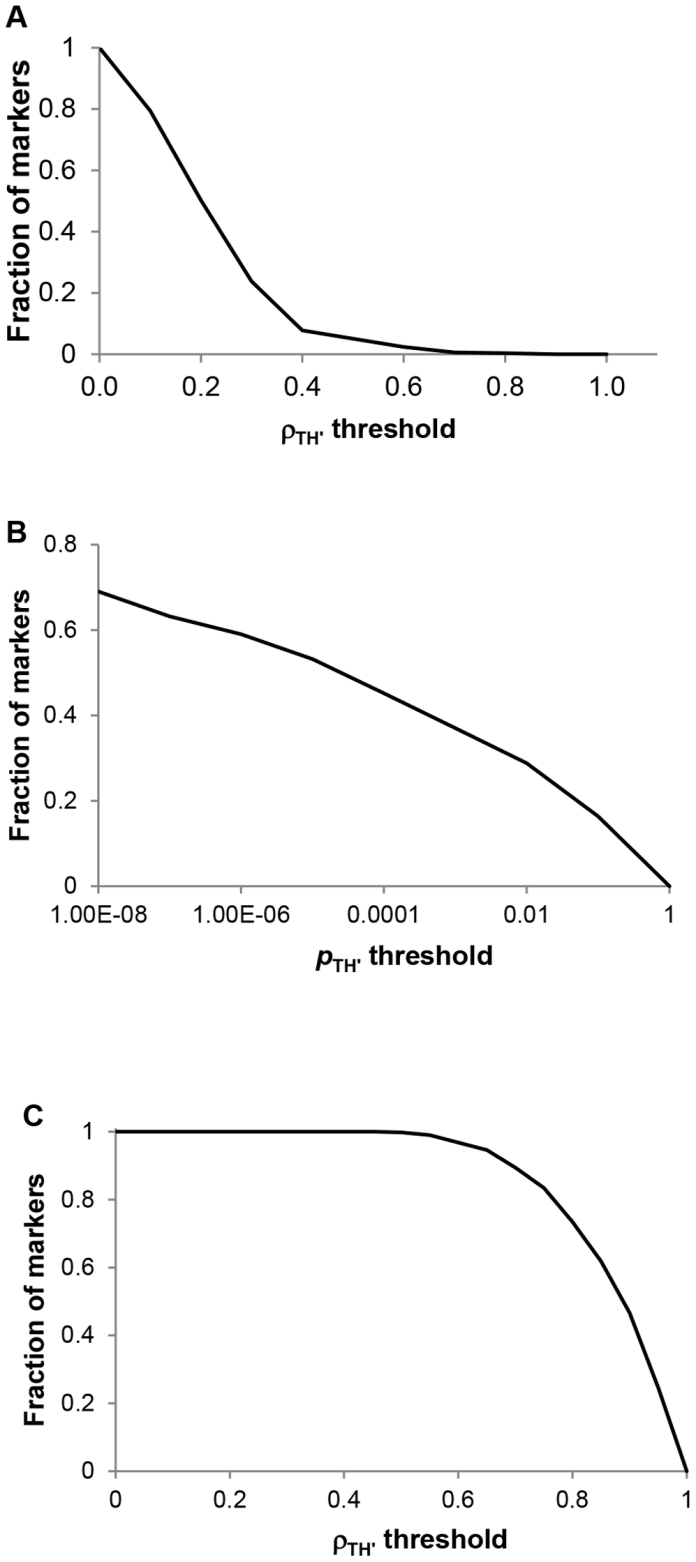
Correlations and 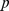-value of markers. A) Fraction of markers satisfying 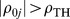. B) Fraction of markers whose 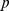-value is greater than 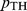. C) Fraction of markers with 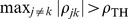.



Figure 3A shows the fraction of markers whose correlation with the ILI data is greater than a prescribed value, 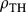. It is observed that there are about 50% of the markers whose correlation with the ILI data is less than 0.2. In Figure 3B, the fraction of markers whose 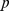-value is less than 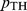 is plotted. It was shown that more than 60% of the markers have 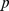-value greater than 0.01. In order to further see the characteristics of the markers, we counted the number of markers whose correlation with other markers is significant. To that end, we plotted 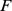 vs. 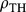, according to

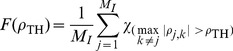
(13)where 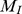 is the number of initial markers and 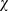 is indicator function. 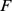 gives the fraction of markers whose correlation with at least one other marker is greater than 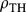. More than 50% of the markers are correlated with at least one other marker with correlation greater than 0.5 (Figure 3C).

### Marker Selection

We observed that there is a significant inter-dependence among the marker frequencies and that frequencies of a considerable number of markers are rather weakly correlated with KCDC’s ILI data. In order to reduce the number of markers for reduced computation, the LASSO method was chosen as it has the advantage of producing sparse solutions, i.e., it will discard candidate features which are proven to be redundant in terms of predictability [13]. LASSO performs the same optimization as the linear regression with an added constraint that the sum of absolute value of the regression coefficients is upper bounded by a tuning parameter 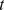. That is, given the desired number of selected markers, 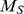, equation (5) described in Methods is modified as follows:
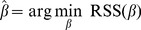
(14)

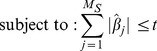
where the RSS is a function of vector of regression coefficients 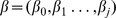, and 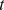 is adjusted such that the number of nonzero 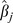
’s is 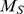.

**Figure 4:**
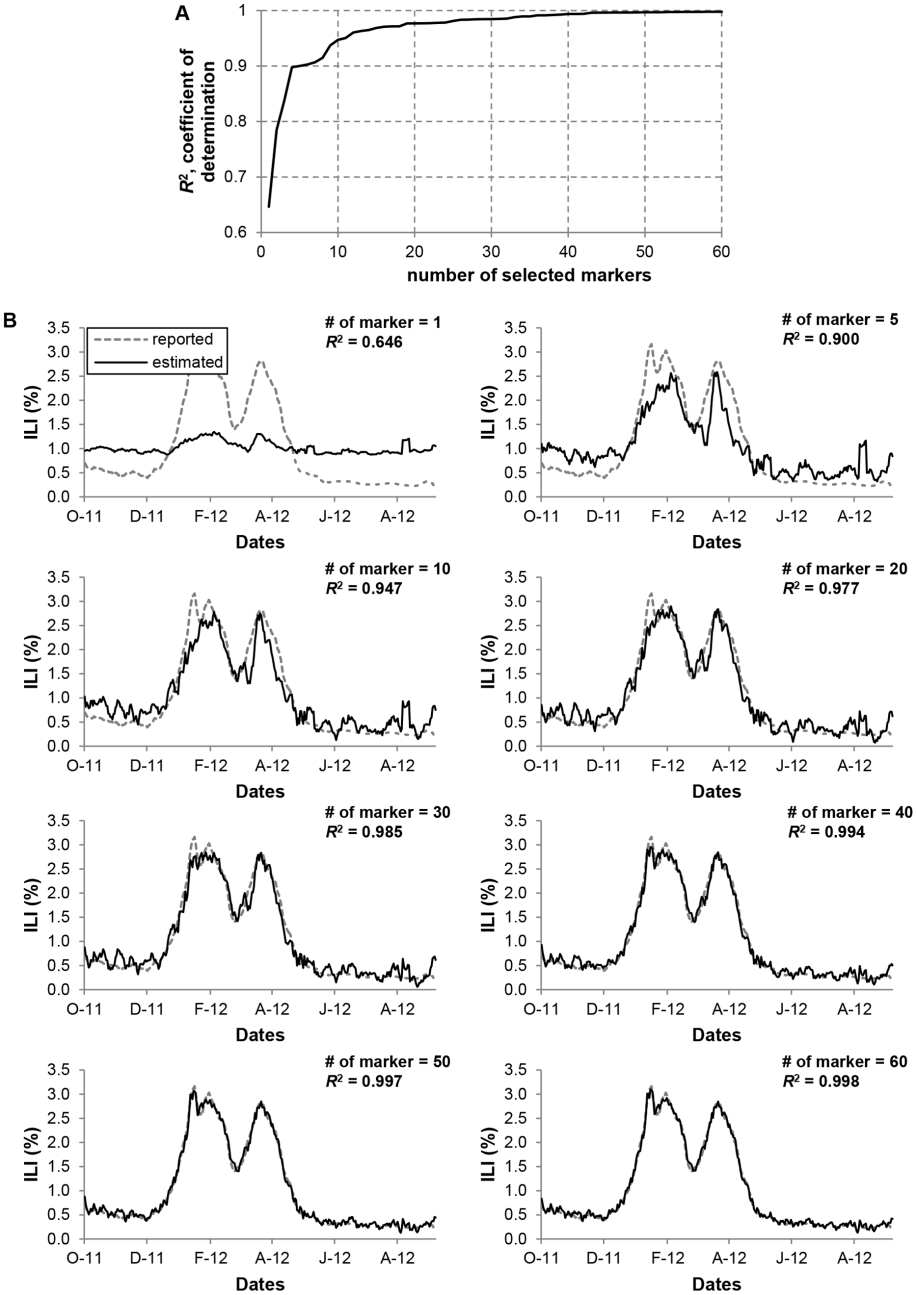
Cross-correlation among the markers. A) The effect of reducing number of markers. 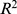 is plotted as a function of number of markers. B) Comparison of 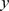 and 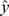. The improvement of the estimation becomes less significant when 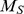 is increased beyond 40.

To see the effect of reducing the number of markers, the coefficient of determination, 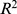, versus the number of selected markers, 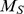, was plotted to find the suitable subset of selected markers (Figure 4A & Table S2). 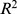 is defined as
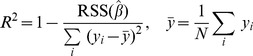
(15)


Thus, 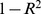 is the normalized RSS. The coefficient of determination can also be seen as the sample correlation between 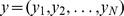 and its estimated sequence 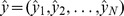. That is,
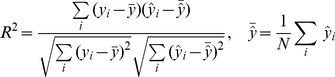
(16)


As we increased the number of selected markers, 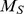, starting from 1, 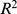 initially increased rapidly, followed by a plateau or levelling out of its value (Figure 4A & Table S2). When the number of markers 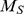 is greater than 30, 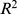 reached a plateau, close to 1.

At this point LASSO algorithm was used to generate subsets of selected markers and their regression coefficients which maximise the correlation with the ILI data, while minimising the size of the marker set. Notably, Figure 4B shows the comparison between 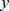 and its estimate 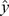 for a number of selected markers, 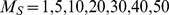 and 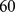. The linear regression gave an excellent estimation of the KCDC’s ILI with coefficient of determination in excess of 0.99 with 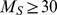. The visual inspection of the figures confirm the earlier observation that the improvement of the estimation becomes less significant when 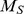 is increased beyond 40. For the remainder of this paper, we will use 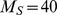 and re-index 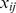 such that ‘
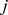
’ denotes the 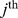 marker selected by LASSO.

### ILI Prediction

**Figure 5:**
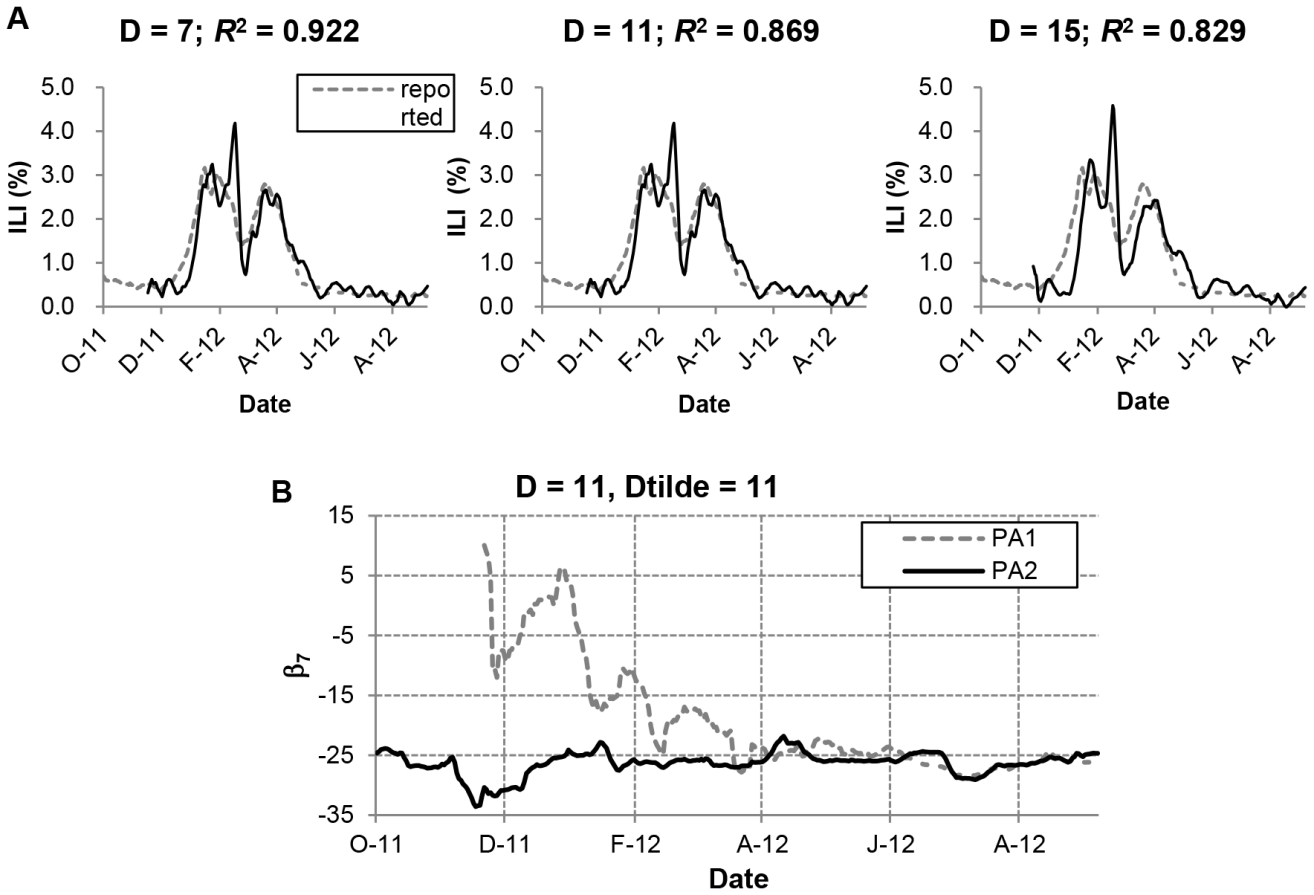
Estimation in the absence of ILI data. A) PA1: comparison of 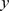 and 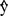 for 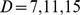. The prediction error increases as 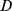 increases. B) Comparison of regression coefficients. 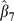 versus days is plotted.

The above analysis assumes that the entire ILI data reported by the KCDC is available at the time of estimation. There is typically a one to two-week delay between the time a patient is diagnosed and the moment that data points become available in ILI reports. Accordingly, when we used prediction algorithm PA1, it was found that this algorithm was implementable for prediction of the ILI despite reporting delay of KCDC’s ILI and could be used under the environment when the model is ‘slowly’ time varying. However, the use of this algorithm resulted in a significant estimation error during the beginning of winter seasonal peak, whereas this gave a fairly good prediction during the middle and latter part of the influenza season (Figure 5A). The comparison of the predicted values and the KCDC’s ILI for three different values of KCDC’s report delay, 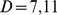, and 15, demonstrated the inability of the prediction to follow the initial rise of the ILI data. It was soon realized that the prediction error increased as 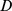 increased. At the beginning of a season when ILI data for computing regression coefficients was not sufficient, the prediction tended to be significantly different from the reported KCDC’s ILI data.

In order to see the underlying mechanism of this error, one of the regression coefficients, say 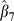 versus days was plotted in Figure 5B, with the value of the same coefficient obtained assuming that the entire KCDC’s ILI data as well as the marker frequency matrix is available. This figure showed that computation of 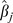
’s based on data on the set of days 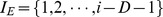 gives considerably different value when ‘
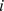
’ (index representing day of the season) is less than 100. However, as ‘
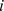
’ increasesd, the discrepancy between these two became less pronounced.

**Figure 6:**
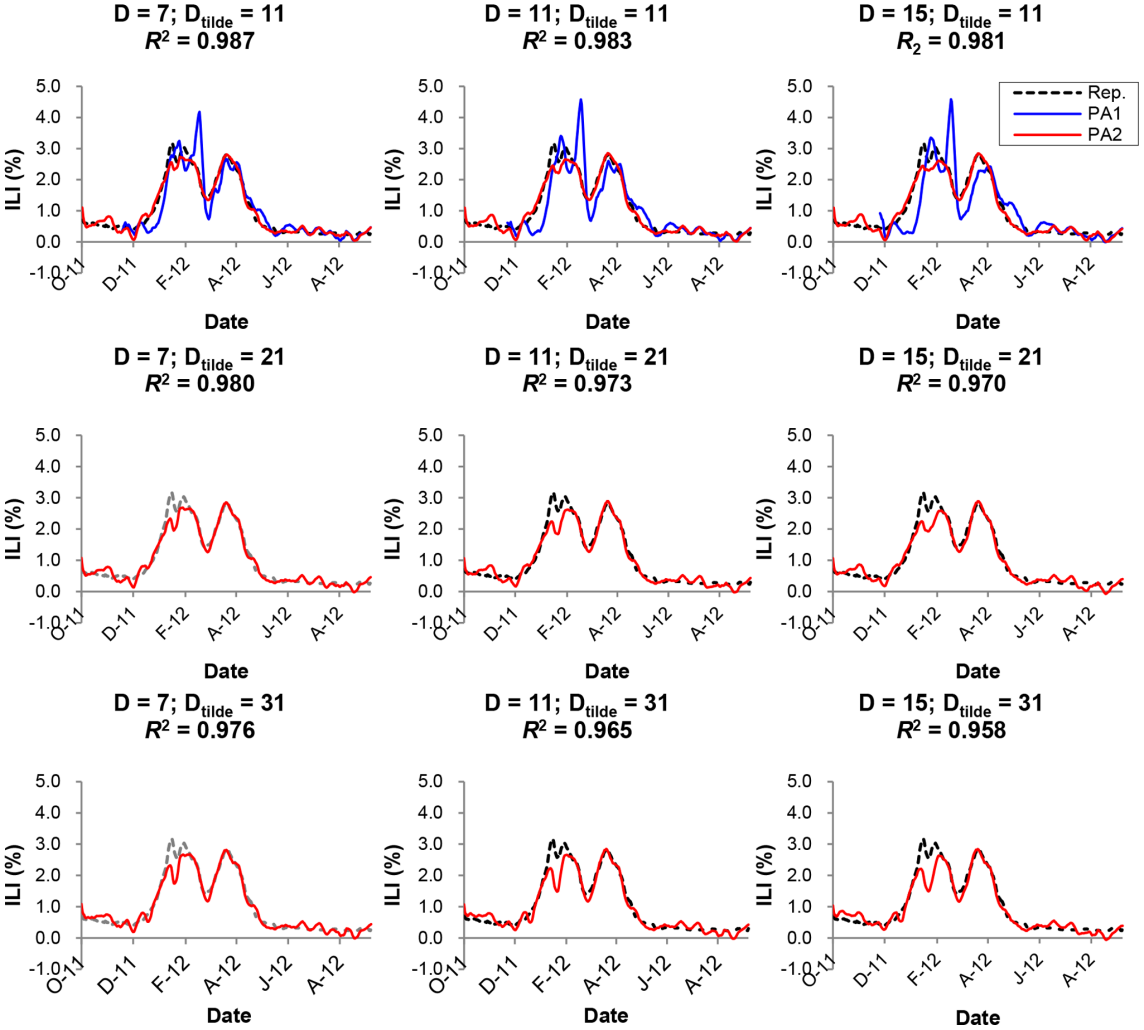
PA2: Comparison of 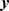 and 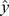. The predicted values of ILI are compared with those reported by the KCDC for 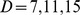 and 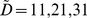. The curves obtained using PA1 (blue lines) were superimposed for comparison. A) 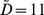 and 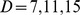. B) 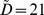 and 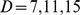. C) 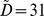 and 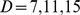.

In Figure 6, we compared the predicted values of Twitter data obtained using PA2 with those reported by the KCDC for 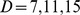 and 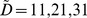. For comparison, we duplicated the results obatined using PA1. It was shown that the modified algorithm, PA2, gave a considerably better prediction for 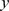 than PA1 with the parameter values considered. The improvement was paricularly significant during the initial period of ILI rise when PA1 had difficulty in predicting. The prediction error, however, increased somewhat as the post blackout period 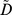 increased. Taken together, even during the beginning of winter seasonal peak, predictive regression algorithms in our model obtained a reasonably reliable prediction of the ILI data.

**Figure 7:**
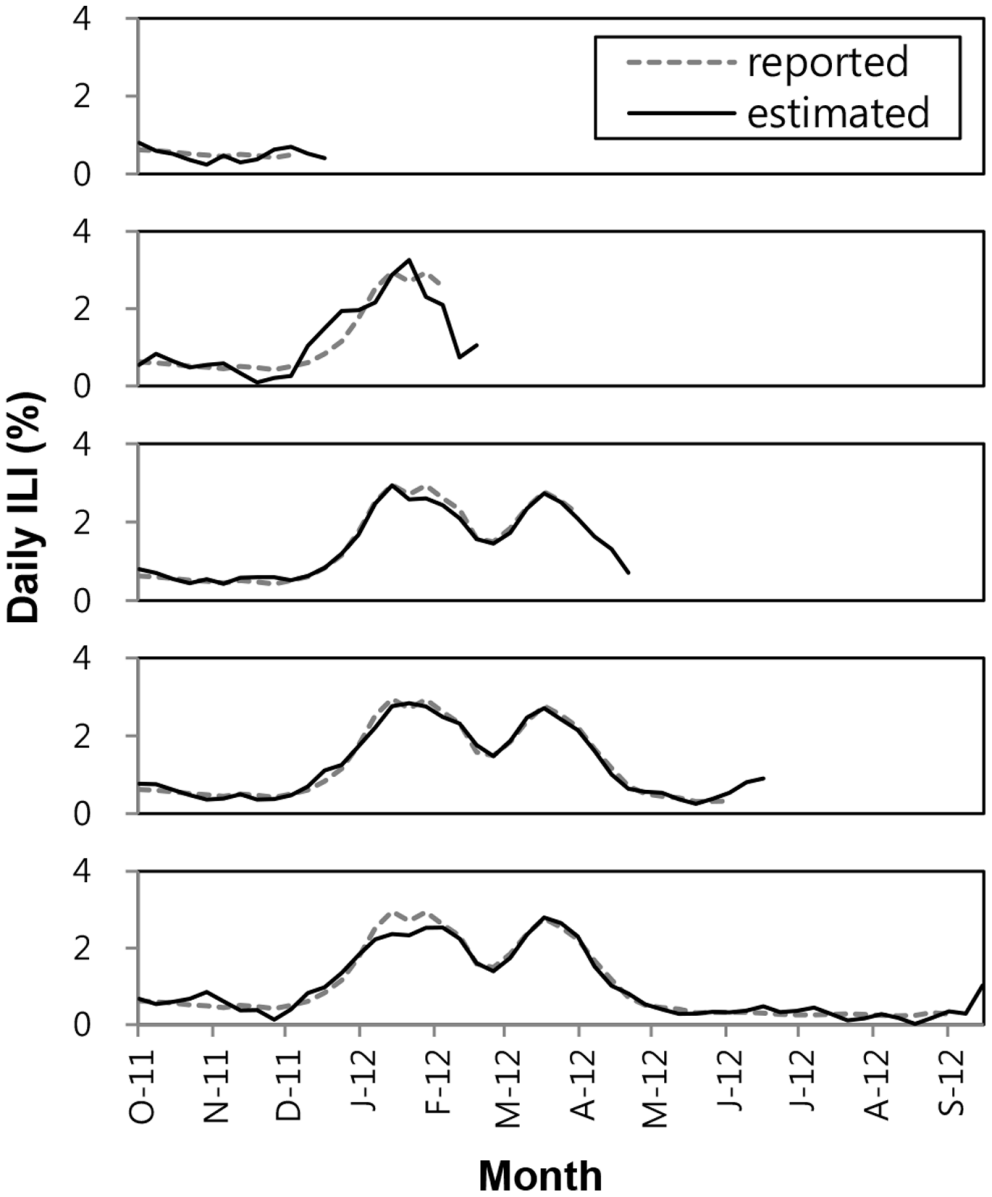
ILI percentages predicted by our model and provided by the KCDC for each month between Dec. 2011 to Sep. 2012. The data were compared at 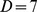 and 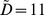.

Throughout the 2011-2012 influenza season we used our model using the modified algorithm PA2 to generate ILI estimates to evaluate timeliness and accuracy of the delay mode. Figure 7 illustrates data available at different points throughout the season for 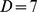 and 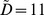. During the time course of the season, we were able to estimate not only the current ILI percentage 1-2 weeks ahead of the reports by KCDC but also a predictive ability of our model.

## Discussion

Despite substantial progress in many areas of influenza research, how and when a new influenza virus strain emerges and spreads rapidly remain largely unknown. Seasons with higher influenza mortality are associated with higher disease transmission and more rapid spread than are mild ones. Although influenza reoccurs each season in regular cycles, efforts to produce reliable and timely estimates of influenza activity are complicated. Various forecasting methods for ILI, using telephone triage calls [15], over-the-counter medications for respiratory diseases [16]–[19], school absenteeism [20], and digital media [2]–[4] have yielded information about future influenza activity for days to weeks in advance of ILI surveillance. Twitter data can monitor the disease activity faster than current practice allows. Our results demonstrate that Twitter data can be used to track and estimate users’ concerns related to influenza disease activity in real time.

In this study, daily influenza spreading score was estimated using linear regression algorithm with ILI data and LASSO selected marker frequency matrix, and the linear correlation coefficients between the tweet and ILI data were used as the performance indicator. Our results revealed important features that make a significant contribution to the goodness-of-fit of the regression models. First, the LASSO method was chosen to select a subset of markers and their weights to maximize the correlation with the ILI data. Although the more marker terms were included the better performance was achieved in terms of correlation coefficients, there was a strong inter-dependence among the marker frequencies. Optimization of the number of markers was necessary and subsequent automatic marker selection generated a set of 40 markers (Table 1) [21]. Linear regression revealed large positive coefficients assigned to markers like ‘novel flu’ (+4.743), ‘lightly’ (+2.668), ‘flu’ (+2.277), and so on. There were also markers with large negative coefficients like ‘surely’ (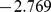), ‘winter’ (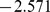), ‘concert’ (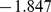) and so on. While our model from a large set of markers can overfit, about 40 selected markers were found to be sufficient to obtain reliable results.

**Table 1:** Selected 40 markers and their linear regression coefficients.

Index	Marker^1^	Pronunciation with Hangeul^2^	Regression coefficient
1	novel flu	[sin.N.p h l.ru ]	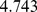
2	surely	[s@l.ma]	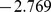
3	lightly	[ja:lp.da]	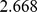
4	winter	[kj@.ul]	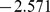
5	flu	[p h l.ru]	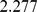
6	concert	[koN.j@n]	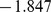
7	similar	[kat.s p.ni.da]	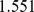
8	for mercy’s sake	[t@k.bun.e]	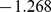
9	once	[il.tan]	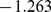
10	severe	[tok.k ha.da]	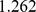
11	pleased	[c l.g@.un]	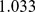
12	serious	[sim.han]	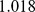
13	eat	[t.si.go]	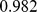
14	autumn	[ka. l]	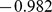
15	recover	[nat.da]	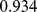
16	become sick	[k@l.ri.da]	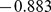
17	shortly	[k m.baN]	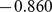
18	tweet	[t h.wit]	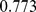
19	cough	[ki.c him]	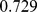
20	injection	[cu.sa]	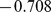
21	fighting	[p ha.i.t hiN]	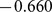
22	by the way	[k n.de]	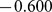
23	good night	[kut.bam]	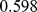
24	live	[ci.nE.da]	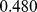
25	realize	[al.at.da]	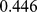
26	give	[cu.sib.si.jo]	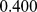
27	condition	[k h@n.di.sj@n]	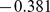
28	birthday	[sEN.il]	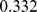
29	early	[c ho.gi]	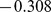
30	air conditioner	[e.@.k hEn]	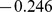
31	transfer	[po.nE.da]	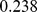
32	severe	[tok.k hE.jo]	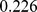
33	dog	[kE]	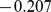
34	noze	[k ho]	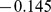
35	actually	[i.man]	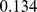
36	anyway	[an.g.rE.do]	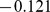
37	be	[ib.ni.da]	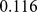
38	almost	[k@. y]	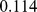
39	have a rest	[swi.da]	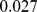
40	haha	[ha.ha]	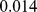

1 Hangeul markers translated to English.

2 Symbols from the international phonetic alphabet (IPA) [21].

Second, linear regression was implemented to examine the prediction performance of our model. Computation of the coefficients depended largely on KCDC`s ILI data when its value was fully available. However, since ILI surveillance reports come via identification of influenza viruses with a delay of up to 1–2 weeks, direct comparison of the predicted values with the ILI data was not straightforward. In the absence of KCDC’s most recent ILI data, prediction resulted in a significant error at the beginning of the influenza season. This is due to insufficient training data set at the beginning of the current influenza season if only the current season’s data were used. This is understandable because computation of the regression coefficients using equations of (9) was based on data collected during the time when ILI is near baseline value. If the previous influenza seasonal data are available, we can use them to improve the accuracy of prediction especially during the period when our algorithm suffers from significant errors. We noted that there are clear seasonal variations in the occurrence of influenza, with a marked peak at wintertime in temperate regions. Therefore, the problem can be circumvented by using data accumulated during the previous influenza season. In order to ensure the fitting algorithm converging despite this problem, we assumed that previous year’s seasonal data are very similar to the present year’s with regard to the seasonal patterns and applied the same algorithm. However, we avoided using the data in the neighbourhood of the 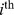 day by introducing a post blackout period 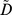 in addition to the report delay 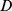. Our experiment with duplicated data demonstrated considerable improvement of prediction accuracy.

Annual epidemics of influenza typically occur during the winter months, but the peak of influenza activity can occur in late spring. Influenza activity recently tends to reach two peaks a year in East Asia. Regardless of the peak time of influenza activity, however, when the KCDC’s ILI data of the previous season is available, our algorithm is expected to lead to a considerably more accurate prediction for seasonal influenza activity than when the previous seasonal ILI data is absent. This is a significant improvement in prediction performance, since it is achieved at the initial phase of ILI peak, when prediction is of most importance and enough data of the present season are not yet available to accurately perform the prediction. Moreover, by calculating the regression coefficients by using the most recent set of data, our algorithm can easily adapt to time-varying environment, albeit, slowly. Nevertheless, our algorithm has a few limitations at present. Our data analysis has been restricted to a single season of epidemic influenza in a single location. It was recently found that Google tracking may not work well for diseases with considerable media exposure, in particular, emerging diseases such as 2009 pandemic H1N1 or severe acute respiratory syndrome [22].

In conclusion, we proposed an adaptive algorithm for real-time prediction of influenza infection using Hangeul Twitter. The feasibility of using the algorithm for a real-time assessment of the current epidemic condition was demonstrated by a reasonablly good prediction of influenza seasonal activity.

## Supporting Information

Table S1 **500 most common words.**
(PDF)

Table S2 **R^2^ difference with increasing numbers of selected markers.**
(PDF)
