# Supplementary material for: Use of Hangeul Twitter to Track and Predict Human Influenza Infection
Source: PLoS One. 2013 Jul 24;8(7):e69305. doi: 10.1371/journal.pone.0069305 (PMC3722273; doi:10.1371/journal.pone.0069305)
Supplement: Table S1 — 500 most common words. (PDF) [file pone.0069305.s001.pdf]

Table S1. 500 most common words set

Marker[1]	Pronunciation with Hangeul[2]
a day	[ha.ru]
a little	[jak.gan]
a long time	[o.rɛ], [o.rɛn]
a minute ago	[a.ka]
abnormal	[i.saŋ]
absolutely	[ɟəl.dɛ]
ache all over	[mom.sal]
actually	[i.man]
afternoon	[o.hu]
again	[ta.si]
ah-ah	[e.ə.kʰɛn]
air conditioner	[mo.du]
all	[nɛ.nɛ]
all the time	[kə.ɨy]
almost	[hon.ɟa]
alone	[pʰəl.s’ə]
already	[i.mi], [jək.si]
also	[tal.go]
always	[haŋ.saŋ], [a.mu]
any	[am.tʰɨn]
anyhow	[an.gɨ.rɛ.do]
anyway	[kɨ.njaŋ]
as it is	[kɨ.t’]
at that time	[ka.ɨl]
autumn	[ib.ni.da]
back	[it.go]
be	[it.ne], [it.nɨnəde], [it.da], [it.sɨp.ni.da], [it.ə.jo], [it.ət.da], [it.ɨ.mjən], [itɨl.ka], [it.sɨm], [it.ɟi], [ta.njə.o.da], [ɟuk.get.ne]
because of	[ɟal.ha.go]
become	[kəl.rye], [kəl.ryet], [kəl.rin], [kəl.rim]
become sick	[t’ɛ.mun], [tø.ge], [tø.nɨn], [tø.mjən], [tø.se.jo], [kəl.ri.da]
best	[kəl.ril]
beware	[ka.ɟaŋ]
birthday	[ɟo.sim]
body	[sɛŋ.il], [əŋ.əŋ], [mom.do], [mom.e], [mom.ɨn]
by the way	[mom.ɨl]
bye	[mom.i]
called	[hɨk.hɨk]
care of oneself	[kɨn.de]
careful	[pʰa.i]
cheer	[ra.go]
cheer up	[mom.ɟo.ri]
children	[ɨŋ.wən], [him.nɛ]
chilly	[a.i]
clothes	[ɛ.dɨl]
cold	[s’al.s’al], [ot.cʰɨl], [cʰu.u.ni.ka], [cʰu.un], [cʰu.wə], [cʰu.wi], [cʰup.go], [cʰup.ne], [cʰup.da], [cʰup.sɨ.ni.da]

Table S1. 500 most common words set -*continued*

Marker[1]	Pronunciation with Hangeul[2]
come	[c^hup.ɟjo], [c^hup.ɟi], [o.go], [o.nɨn], [wa.sə], [wat.sə.jo]
common cold	[na.o.go]
complete	[na.wa.sə]
concert	[kam.gi]
condition	[wan.ɟən], [koŋ.jən]
consecutively	[saŋ.t^hɛ]
consequence	[k^hən.di.sjən]
continually	[nɛ.ri]
control	[na.ɟuŋ]
cough	[kje.sok], [kwan.ri]
cover	[ki.c^him]
crazy	[k^hol.rok]
crying	[təp.go], [mi.c^hin]
daily temperature difference	[il.gjo.c^ha]
damn	[ɟon.na]
dawn	[sɛ.bjək]
daytime	[nat.e]
degree	[ɟəŋ.do]
delicious	[mat.it.da]
different	[ta.rɨn]
discharge	[ɟe.dɛ]
dislike	[sil.t^ha], [silh.ə]
do	[ha.go], [ha.na], [ha.ne], [ha.nɨn], [ha.ni], [ha.da], [ha.mjən], [ha.se.jo], [ha.si,go], [ha.ɟi.man], [han.da], [hap.ni.da], [hɛ.do], [hɛ.sə], [hɛ.ja], [hɛ.jo], [hɛt.nɨn.de], [hɛt.də.ni], [hɛt.ə.jo]
do not	[hɛ.ɟu.se.jo], [mo.rɨ.get.da], [mol.ra], [kɛ]
do not know	[ma.se.jo], [mal.go], [mal.a]
dog	[an.dwɛ]
drink	[an.dø.da]
dying	[ma.si.go]
eagerness	[jəl.sim]
early	[mən.ɟə], [il.c'ik], [c^ho.gi]
eat	[tɨ.se.jo], [tɨ.si.go], [mək.go], [mək.ə], [mək.ət.da], [mək.ɨ.mjən]
effort	[su.go]
elder brother	[o.p'a], [o.p'an]
elder sister	[ən.ni]
enter	[tɨl.ə.ga.da]
especially	[t^hɨk.hi]
even so	[kɨ.na]
evening	[ɟə.njək]
everyday	[mɛ.il], [mɛn.nal]
everyone	[ta.dɨl]
examination	[si.həm]
exercise	[un.doŋ]
extremely	[mu.ɟi]

Table S1. 500 most common words set -*continued*

Marker[1]	Pronunciation with Hangeul[2]
eyes or snow	[nun.ɨn], [nun.ɨl], [nun.i]
facial	[əl.gul]
fact	[sa.sil]
fairly	[ɟe.bəp]
fatigue	[p^{h}i.gon]
favor	[ki.bun]
feeling	[nɨ.kim], [jəl.i]
fever	[a.ɟa]
fighting	[p^{h}a.i.t^{h}iŋ], [hwa.i.tiŋ], [hwat.tiŋ], [c^{h}wal.jəŋ]
filming	[kjəl.guk]
finally	[tɨ.di.ə], [kwɛn.c^{h}an.a]
fine	[an.jəl.ri.da], [ɟot.get.da], [ɟot.t^{h}a], [ɟot.a.jo], [ɟoh.ɨn], [ɟot.c^{h}i], [p^{h}ɨl.ru]
flu	[sən.pal]
following	[wi.hɛ]
for	[ɟam.k'an]
for a little while	[na.han.te]
for me	[tək.bun.e]
for mercy's sake	[ta.hɛŋ]
fortunate	[ɟi.dok]
foul	[ɟa.ɟ.u]
frequently	[c^{h}in.gu]
friend	[k'oŋ.k'oŋ]
frozen hard	[ke.da.ga]
furthermore	[il.ə.na.da]
future	[na.a.ra]
get up	[na.at.da]
get well	[na.ɨ.mjən], [na.ɨl], [ɟu.se.jo], [cu.sib.si.jo]
give	[ka.sə], [t'əl.ə.ɟjə]
go	[ta.ni.da], [ka.go], [kat.t秀召ˋa], [kut.mo.niŋ]
go away	[kut.na.it]
good	[kut.bam]
good morning	[ɟal.ɟa]
good night	[ɟu.mu.se.jo], [ɟəm.ɟəm], [hɛŋ.bok], [him.dɨl.da]
gradually	[ko.sɛŋ]
ha-ha	[sy.go]
happy	[swi.da]
hard	[sy.se.jo]
hardships	[sy.ə]
have a rest	[ha.ha], [mə.ri], [tu.t^{h}oŋ], [tɨt.go]
head	[jə.gi]
headache	[jə.gin]
hear	[an.njən]
here	[ɟip.e], [p^{h}jəŋ.wən]
hi	[tə.un]
home	[tə.wə]
hospital	[təb.go]

Table S1. 500 most common words set -*continued*

Marker[1]	Pronunciation with Hangeul[2]
hot	[ə.t'ɛ], [ə.t'ət.ge], [əl.ma]
how	[sa.ram], [na.nɨn], [nɛ.ga]
human	[ɟə.nɨn]
I	[ɟe.ga], [əl.ɨn], [mjən.jək], [ap.ɨ.ro]
immediately	[ɟən.e]
immunity	[mot.ha.da]
inferior	[tok.gam]
influenza	[ɟu.sa]
injection	[tɛ.sin]
instead	[ɨŋ.ɨŋ]
jackpot	[tɛ.bak]
japan	[il.bon]
korea	[han.kuk]
last	[ma.ji.mak]
lately	[cʰø.gɨn]
laugh	[ut.sɨm]
lightly	[sɨl.sɨl], [jaːlp.da]
like this	[i.rə.go], [i.rən], [i.rəl.tʰɛ], [i.rət.ge]
live	[ci.nɛ.da]
love	[sa.raŋ]
many or much	[ma.ni], [manh.a.jo], [manh.ɨn], [manh.i]
me	[na.rɨl]
me too	[na.do], [na.du], [ɟə.do], [ɟə.du]
medicine	[jak.do], [jak.ɨn], [jak.ɨl]
meet	[man.na.da]
mention	[men.sjən]
MiKoo	[mi.kʰu]
mind	[ma.ɨm], [ɟəŋ.sin]
mom	[əm.ma]
morning	[a.cʰim]
most	[ɟe.il]
my	[na.ɨy]
nasal discharge	[kʰot.mul]
naturally	[wən.rɛ]
near	[jəp.e]
neck	[mok]
new year	[sɛ.hɛ]
next	[ta.ɨm]
nice	[mət.jin]
night	[pʰam.e]
nose	[kʰo]
not	[a.nja], [a.ni], [a.nin], [a.nim], [anh.ge], [anh.go], [anh.nɨn], [anh.do.rok], [anh.a.jo], [əp.go], [əp.nɨn], [əp.da], [əp.sə], [əp.si]
novel flu	[sin.ɟoŋ.pʰɨl.ru]
now	[i.ɟe], [i.ɟen], [ɟi.gɨm]
nowadays	[jo.sɛ], [jo.sɟɨm]

Table S1. 500 most common words set -*continued*

Marker[1]	Pronunciation with Hangeul[2]
oh-oh	[a.a], [a.o]
once	[il.tan], [han.bən]
one by one	[c^{h}ak.c^{h}ak]
one week	[il.ɟu.il]
our	[u.ri]
out	[p^{h}ak.e]
particularly	[p^{h}jəl.ro]
past	[a.ma]
perhaps	[p^{h}un.dɨl]
person	[sa.ɟin]
photo	[ɟe.bal]
please	[cɨl.gə.un]
pleased	[ɟɨl.gəp.da], [je.baŋ]
prevent	[hok.si]
probably	[ka.nɨn]
progress	[p'al.ri]
quickly	[ə.sə], [ə.jə], [ən.nɨŋ], [i.bul]
quilt	[p^{h}i.ga]
rain	[p^{h}i.o.nɨn]
raining	[ɟin.c'a]
real	[al.at.da]
realize	[ɟəŋ.mal]
really	[nat.da]
recover	[jə.ɟən]
remain unchanged	[ɟa.k'u]
repeatedly	[nam.ɨn]
rest	[mat.go]
right	[mat.a.jo], [mal.i]
say	[mu.sə.wə]
scare	[hak.kjo]
school	[p^{h}o.go]
see	[p^{h}o.ni], [p^{h}o.da], [p^{h}o.mjən], [p^{h}wa.jo], [sə.ul]
seoul	[sim.han]
serious	[sim.hɛ], [əm.c^{h}əŋ]
seriously	[jə.rə]
several	[mjə.c^{h}il]
several days	[tok.k^{h}a.da]
severe	[tok.han], [tok.k^{h}ɛ.jo], [sim.ha.da], [mo.sɨp]
shape	[kɨm.baŋ]
shortly	[a.p^{h}a]
sick	[a.p^{h}ɨ.da], [a.p^{h}ɨn], [kat.ne.jo]
similar	[kat.t秀召ˋa], [kat.sɨp.ni.da], [ka.ta.yo], [ka.tɨn], [kat.i], [ɟin.sim]
sincerely	[k^{h}o.gam.gi]
sinus cold	[nim.do]
sir or ma'am	[nu.na]
sister	[ɟa.go]

Table S1. 500 most common words set -*continued*

Marker[1]	Pronunciation with Hangeul[2]
sleep	[ɟa.da], [ɟa.myən], [ɟa.ja.ɟi], [ɟa.jo], [ɟam.do], [ɟam.ɨl], [ɟam.i], [ɟol.rjə]
sleepy	[sal.c'ak]
slightly	[hul.c'ək]
sniffle	[kɨ.rɛ]
so	[wɛ.i.ri], [ɟo.gɨm]
some	[wɛn.ji]
somehow	[mwən.ga]
something	[no.rɛ]
song	[mok.gam.gi]
sore throat	[mok.a.p^{h}a], [mok.i.a.p^{h}ɨ.da], [mi.an]
sorry	[ɟø.soŋ], [so.ri]
sound	[ki.un]
stamina	[a.ɟik]
still	[p^{h}a.ro]
straight	[kət.do]
stuff	[kət.ɨn], [kət.i], [tɛ.t^{h}oŋ]
success	[kɨ.rə.ge]
such	[kɨ.rən], [kɨ.rəm], [kɨ.rət.c^{h}i], [kɨ.ri], [kap.ɟa.gi]
suddenly	[jə.rɨm]
summer	[səl.ma]
surely	[ɟɨŋ.saŋ]
symptom	[ka.ɟi.go]
take	[c^{h}ɛŋ.gjə], [c^{h}ɛŋ.gi.da], [jak.mək.go]
take medicine	[he.he]
tehee	[hi.hi], [ki.on]
temperature	[ko.map.sɨp.ni.da]
thanks	[kam.sa], [ko.ma.wə], [kɨ.gə]
that	[kɨ.gən], [kɨ.ge], [hwan.ɟəl.gi]
the change of seasons	[kɨ.rə.mjən]
then	[kɨ.rɛ.sə]
therefore	[sɛŋ.gak]
thinking	[i.gə]
this	[i.gən], [i.ge], [i.nom], [i.ri], [i.bən]
this time	[ol.hɛ]
this year	[k'ok.k'ok]
tightly	[si.gan]
time	[o.nɨl]
today	[ham.ke]
together	[nɛ.il]
tomorrow	[nə.mu]
too	[po.nɛ.da]
transfer	[mom.gwan.ri]
treat oneself	[t^{h}ɨ.wit]
tweet	[t^{h}ɨ.c^{h}in]
twitter friend	[u.san]

Table S1. 500 most common words set -*continued*

Marker[1]	Pronunciation with Hangeul[2]
umbrella	[ɨ.ɨ]
urrrr	[kwɛn.hi]
uselessly	[mɛ.u]
very	[a.ɟu], [p^{h}i.t^{h}a.min]
vitamin	[mok.so.ri]
voice	[sip.da]
want	[sip.ə], [sip.ɨn], [t’a.dɨ.han]
warm	[t’a.t’ɨ.han], [t’a.sup.ge], [ip.go]
wear	[ip.gu], [ip.ə.jo], [ip.ɨ.se.jo], [nal.s’i]
weather	[nal.i], [ɟu.mal]
week-end	[ɟu.ɨy.ha.se.jo]
wellness	[kən.gaŋ]
what	[mu.sɨn]
when	[ən.ɟe]
where	[ə.di]
which	[ə.t’ən]
who	[nu.ga]
whole	[mo.dɨn]
wind	[p^{h}a.ram]
winter	[kjə.ul]
wish	[p^{h}a.rap.ni.da], [p^{h}a.rɛ.jo]
work	[il.i]
worry	[kək.ɟəŋ]
wow	[u.wa]
yes	[ə.ɟe]
yesterday	[ɨ.a]
you	[nə.do], [nə.du], [ni.ga], [taŋ.sin]

Supporting Information Legends

[1] Hangeul markers translated to English
[2] Symbols from the international phonetic alphabet
