# Supplementary material for: Use of Hangeul Twitter to Track and Predict Human Influenza Infection
Source: PLoS One. 2013 Jul 24;8(7):e69305. doi: 10.1371/journal.pone.0069305 (PMC3722273; doi:10.1371/journal.pone.0069305)
Supplement: Table S2 — R2 difference with increasing numbers of selected markers. (PDF) [file pone.0069305.s002.pdf]

Table S2. R^2 difference with increasing numbers of selected markers

No. of selected markers	R^2	No. of selected markers	R^2	No. of selected markers	R^2	No. of selected markers	R^2
1	0.64632	16	0.97072	31	0.98478	46	0.99627
2	0.78462	17	0.97130	32	0.98531	47	0.99631
3	0.83696	18	0.97142	33	0.98794	48	0.99634
4	0.89768	19	0.97661	34	0.98923	49	0.99659
5	0.90006	20	0.97667	35	0.98933	50	0.99677
6	0.90261	21	0.97682	36	0.99123	51	0.99681
7	0.90670	22	0.97707	37	0.99127	52	0.99685
8	0.91482	23	0.97787	38	0.99195	53	0.99720
9	0.93714	24	0.97829	39	0.99252	54	0.99726
10	0.94706	25	0.98104	40	0.99373	55	0.99738
11	0.95039	26	0.98340	41	0.99379	56	0.99744
12	0.96044	27	0.98350	42	0.99389	57	0.99748
13	0.96293	28	0.98428	43	0.99603	58	0.99766
14	0.96486	29	0.98442	44	0.99625	59	0.99770
15	0.96860	30	0.98447	45	0.99626	60	0.99775
